# In vitro antidiabetic effects of selected fruits and vegetables against glycosidase and aldose reductase

**DOI:** 10.1002/fsn3.243

**Published:** 2015-04-30

**Authors:** Tong Wu, Jiaqiang Luo, Baojun Xu

**Affiliations:** ^1^ Food Science and Technology ProgramBeijing Normal University‐Hong Kong Baptist University United International CollegeZhuhaiGuangdong519085China

**Keywords:** Aldose reductase, antioxidant properties, diabetics, natural food products, *α*‐glycosidase

## Abstract

In vitro antidiabetic effect of fruits and vegetables with reports as folk remedies were investigated. The antidiabetic effects were evaluated by comparing the inhibitory properties of *α*‐glycosidase, aldose reductase, and antioxidant activity. The results indicated that lychee extract exhibited the best dose‐dependent inhibitory activity against *α*‐glycosidase with IC_50_ of 10.4 mg/mL, and lemon peel extract exhibited aldose reductase inhibitory potential with IC_50_ value at 3.63 mg/mL. Besides, the result also showed that the inhibitory effects of blueberry and plum against *α*‐glycosidase were strong among the fruits samples. Bitter gourd and eggplant demonstrated significant inhibitory potential against aldose reductase, with IC_50_ values at 8.55 mg/mL and 8.06 mg/mL, respectively. The result from correlation analysis part showed that the antioxidant activities of selected fruits and vegetables were found related to their health beneficial effects, as there was positive correlations between total flavonoids content (TFC) and aldose reductase inhibitory activity (*r* ^2^ = 0.556).

## Introduction

Diabetes mellitus, a disease that resulting from the deficiency or resistance to insulin in human body, affects 347 million people around the world and the mortality is more than 80% in many developing countries (Danaei et al. 2011). Efforts are put to find out the ways to cure or minimize expansion of diabetes mellitus; some factors involved in pathogenesis of diabetes have been identified.

*α*‐Glycosidase is an enzyme located in the intestinal brush border, which is responsible for converting oligosaccharides and disaccharides to monosaccharides, which promotes the absorption of carbohydrate and contributes to the increase in blood sugar concentration (Bischoff 1995). The use of *α*‐glycosidase inhibitor can delay absorption of carbohydrate through competitive inhibition, thus subsequently inhibit the hydrolysis of disaccharides and the absorption of glucose (Vichayanrat et al. 2002). Research has shown the antidiabetes and antiobesity effects of *α*‐glycosidase inhibitor (Elbein, 1991). Moreover, acarbose, a recognized *α*‐glycosidase inhibitor, is clinically used as antidiabetes drug. The use of natural source *α*‐glycosidase inhibitor, including plant and microorganism, has attracted attention of scientists (Kumar et al. 2011). The medical values of these natural sources should be well identified.

The accumulation of intracellular sorbitol through polyol pathway has been recognized as an important reason in the developing clinical complications of diabetes, the complication includes cataract and some neurological diseases (Kador 1988). In hyperglycemia condition, the excessive glucose will not only metabolize from glycolysis, but the polyol pathway in which glucose is converted to sorbitol under the catalysis of aldose reductase, and then subsequently convert to fructose by sorbitol dehydrogenase (Robert et al. 2007). Efforts have been made to find the aldose reductase inhibitor to prevent diabetic complications.

The potentially protective effects including antidiabetic, anticardiovascular diseases of the food containing phytochemicals have been identified (Senevirathne et al. 2006). The phenolic compounds can reduce the oxidative stress and inhibit the marcromolecular oxidation, which are beneficial to the health (Pulido et al. 2000). Antioxidants, such as flavonoids, exhibited significant antidiabetic effect by inhibiting the activity of certain enzymes such as aldose reductase (Kato et al. 2009). Moreover, because of the significant roles of oxidation reactions in advanced glycated end products (AGE) formation, the antioxidant poses hypoglycemic effect through anti‐AGE mechanism (Xi et al. 2010). The food with high antioxidant content often recommended to the diabetic patients.

In China, people advocate food therapy, and many foods are believed to have antidiabetic properties. But these folk remedies in treating diabetics have not been scientifically verified and are not even well understood. In this study, the crude extracts were obtained from fruits and vegetables with reports as folk remedies in antidiabetic efficacy. *α*‐Glycosidase and aldose reductase inhibitory activities as well as the antioxidant activities of these selected samples were evaluated.

## Materials and Methods

### Food materials

Fresh fruits and vegetables were purchased from a local market in Zhuhai. The species of the samples were listed in Table 1 with the information on both English common names and Latin scientific names. The edible parts of the samples were used for investigation.

**Table 1:** A list of selected food samples

Samples	Common name	Scientific name	Part used
Fruits			
1	Apricot	*Prunus armeniaca*	Pulp
2	Lychee	*Lychee chinensis*	Pulp
3	Blueberry	*Vaccinium cyanococcus*	Whole fruit
4	Plum	*Prunus salicina*	Pulp
5	Kiwi	*Kiwifruit c.v. hayward*	Peeled pulp
6	Lemon pulp	*Citrus limon*	Pulp
7	Lemon peel	*Citrus limon*	Peel
8	Pear	*Pyrus bretschneider*	Pulp
9	Wolfberry	*Lycium chinensis*	Whole fruit
10	Water melon	*Citrullus lanatusus*	Melon pulp
Vegetables			
11	Lettuce	*Lactuca sativa*	Leaves
12	Cucumber	*Cucumis sativus*	Fruit
13	Red onion	*Allium cepa*	Peeled onion
14	Bitter gourd	*Momordica charantia*	Whole fruit
15	Eggplant	*Solanum melongena*	Whole fruit
16	Celery	*Apium graveolens*	Stem
17	Kelp	*Laminaria japonica*	Leaves
18	Wax gourd	*Benincasa pruriens*	Whole fruit
19	Garlic	*Allium sativum*	Peeled garlic
20	Tomato	*Solanum lycopeersicum*	Whole fruit

### Chemicals

Sodium carbonate, gallic acid, sodium nitrite, sodium hydroxide, vanillin, ethanol, methanol, disodium hydrogen phosphate, sodium dihydrogen phosphate, dipotassium phosphate, potassium dihydrogen phosphate, and Comassie Brilliant Blue (CBB) were purchased from Tianjin Damao Co. (Tianjin, China). Folin‐Ciocalteu reagent, (+)‐catechin, 2‐diphenyl‐1‐picrylhydrazyl (DPPH), *p*‐nitrophenyl‐*α* ‐glucopyranoside, bovine serum albumin (BSA), and DL‐glyceraldehyde were purchased from Shanghai Yuanye Co. (Shanghai, China). Trolox, porcine *α*‐glycosidase, and *α*‐nicotinamide adenine dinucleotide phosphate and *α*‐nicotinamide adenine dinucleotide 2'‐phosphate reduced tetrasodium salt (NADPH) were purchased from Sigma Chemical Co. (St. Louis, MO).

### Sample extraction

The fresh sample (100 g) was crushed using food masher then extracted with 70% of ethanol (1:10 w/v) twice at room temperature for 24 h and 3 h, respectively. The extracts were collected and concentrated under rotary evaporator at 55°C. After evaporation, freeze dryer was applied to remove the moisture from extracts. The dry extracts were stored at −20°C until analysis. The measurements in this study were done in triplicate and the biological activities of food samples were determined at a concentration of 50, 25, 12.5, 5, 2.5, and 1 mg/mL.

### *α*‐Glycosidase inhibition assay

*α*‐Glycosidase inhibitory activity of the samples was evaluated in 96‐well plate based on the method described by Kwon et al. (2008) with slight modifications (Wu and Xu 2014). In 96‐well assays, each well contained 150 *μ*L reaction mixture of 50 *μ*L of sample extract and 100 *μ*L of phosphate buffer (0.1 mol/L, pH 6.9) contained 1 U/mL of *α*‐glycosidase, and the reaction mixture was preincubated at 25°C for 10 min. After incubation, 50 *μ*L of 5 mmol/L *p*‐nitrophenyl‐*α*‐D‐glycopyranoside solution in phosphate buffer (0.1 mol/L, pH 6.9) was added in each well and incubated at 37°C for 5 min. The absorbance before and after the incubation were determined spectrophotometrically at 405 nm using a microarray reader (Thermo Electron Co., Waltham, MA). The inhibitory activity of extracts from samples was compared with the control which contained 50 *μ*L of phosphate buffer (0.1 mol/L, pH 6.9) instead of sample, and acarbose was used as a positive control. The percentage of inhibition was calculated as:

$$\%\;\text{inhibition}\;=\;\frac{\Delta {\text{Abs}}_{\mathrm{control}}\;-\;\Delta {\text{Abs}}_{\mathrm{sample}}}{\Delta {\text{Abs}}_{\mathrm{control}}}\;\times \;100,$$

where,

Abs_sample_ is the absorbance of sample extract.

Abs_control_ is the absorbance of the control.

### Aldose reductase inhibition assay

#### Extraction of aldose reductase from porcine lenses

The methodology was based on Hayman and Kinoshita (Lim et al. 2001) with slight modification by Fujita et al. (2004). The crude porcine aldose reductase (AR) was prepared from the porcine lenses which purchased from the local market in Zhuhai, China. Briefly, 10 lenses (6 g) were homogenized with 30 mL of 0.1 mol/L potassium phosphate buffer (pH 7.09 at 5°C), and the mixture was then centrifuged at 10,000* g* for 20 min under 4°C. After centrifugation, the supernatant was collected and stored at −20°C until use.

#### Determination of protein concentration of porcine AR

The amount of soluble protein in porcine AR was evaluated using Coomassie Brilliant Blue (CBB). Generally, 100 *μ*L of porcine AR was mixed with 5 mL of CBB solution. After 2 min standing, the absorbance was measured using visible spectrophotometer (772s, Precision and Scientific Instrument Co., Shanghai, China) under 595 nm. Standard curve was made by BSA solution with concentration of 200 *μ*g/mL, 400 *μ*g/mL, 600 *μ*g/mL, 800 *μ*g/mL, and 1000 *μ*g/mL. The concentration of soluble protein in porcine AR was calculated based on the standard curve (*r* ^2^ = 0.997) and was expressed as *μ*g/mL.

#### Determination of porcine AR activity and the AR inhibitory activity of sample extracts

AR activity and AR inhibitory activity were determined based on method of Lim et al. (2001) with some modifications. Briefly, 1 mL of the assay mixture contained 700 *μ*L of phosphate buffer (0.1 mol/L, pH 6.2), 100 *μ*L of 100 mmol/L DL‐glyceraldehyde, 100 *μ*L of NADPH (1.5 mmol/L), and 100 *μ*L porcine AR enzyme solution or 100 *μ*L of test sample. The absorbance of assay mixture was measured against control (contained all the components except for the enzyme) after 10 min incubation at 37°C under 340 nm, using UV–visible spectrophotometer (TU‐1901, Puxi Co. Beijing, China). The activity of porcine AR and AR inhibitory activity of test sample were calculated by following the equations:

$${\text{AR}}_{\mathrm{activity}}\;=\;\frac{{\text{Abs}}_{\mathrm{AR}}\;-\;{\text{Abs}}_{\mathrm{control}}}{{\text{Abs}}_{\mathrm{control}}\;\times \;V_{\mathrm{AR}}}\;\left(\text{expressed in U/mL}\right),$$

$$\%\;\text{inhibition}\;=\;\left[1-\;\left(\frac{{\text{Abs}}_{\mathrm{sample}}\;-\;{\text{Abs}}_{\mathrm{control}}}{{\text{Abs}}_{\mathrm{control}}}\right)\right]\;\times \;100,$$

where,

Abs_AR_ is the absorbance of AR solution,

Abs_control_ is the absorbance of the negative control,

V_AR_ is the volume of AR solution (mL).

### Determination of total phenolic content

This method basically followed the method of Singleton and Rossi (1965) with slight modification by Xu and Chang (2007). The result was presented as gallic acid equivalents (mg gallic acid equivalents/g sample extract) with the calibration curve of gallic acid which had a linearity range from 10 to 1000 *μ*g/mL (*r* ^2^ = 0.999).

### Determination of total flavonoids content

The method followed a previous study (Heimler et al. 2005). The result was presented as catechin equivalents (mg of catechin equivalents/g sample extract) with the calibration curve of catechin which had a linearity range from 10 to 1000 *μ*g/mL (*r* ^2^ = 0.997).

### Determination of DPPH free radical scavenging activity

The methodology was based on the previous study by Chen and Ho (1995) with slight modification. Briefly, 200 *μ*L of sample extract and 3.8 mL of DPPH (0.1 mmol/L) in ethanol were mixed in a test tube. The mixture was vortexed for 1 min, and then put in dark for 30 min. After that, the absorbance was measured at 517 nm with a visible spectrophotometer (772s, Precision and Scientific Instrument Co., Shanghai, China). A negative control which added 200 *μ*L of ethanol solution instead of sample extract was taken. The DPPH scavenging rate was calculated according to the equation below and the IC_50_ (half maximal inhibitory concentration) can be also calculated:

$$\begin{array}{c} \text{DPPH free radical scavenging rate}\;(\%)\\ \;=\left[1-\;\left(\frac{{\text{Abs}}_{\mathrm{sample}}}{{\text{Abs}}_{\mathrm{control}}}\right)\right]\;\times \;100, \end{array}$$

where,

A_sample_ is the absorbance of sample extract or standard solution,

A_control_ is the absorbance of the negative control,

### Statistical analysis

Results were expressed in mean ± standard deviation (SD) of triplicate assay. The statistical analyses of data were performed using the Microsoft Office Excel (2010) and SigmaPlot (12.0). Significant differences among data were analyzed by SPSS Statistic 20 and significance was accepted at level of *P *< 0.05. Pearson's correlation test was conducted to determine the linear correlations among variables.

## Results

### *α*‐Glycosidase inhibitory effects of fruits and vegetables

**Figure 1:**
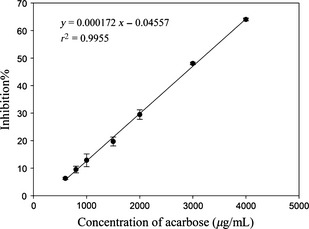
*α*‐Glycosidase inhibitory effect of positive control (acarbose).

**Figure 2:**
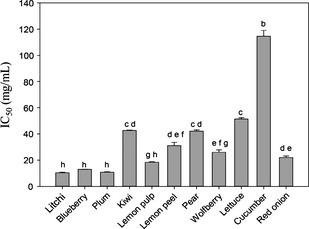
*α*‐Glycosidase inhibitory activity of sample extracts. Figures in the graph are IC_50_ (mg/mL) and are lower than 200 mg/mL (Bitter gourd with an IC_50_ value at 336.7 mg/mL was not shown in this graph). The bars marked by the same letters are not significantly (*P *<* *0.05) different.

The ethanol extract of 20 samples were dissolved into buffer and subjected to *α*‐glycosidase inhibitory assay. The result might relate to the potential antidiabetic activity. The inhibitory activities of sample extracts were shown in Table 2 in terms of IC_50_ values, and the higher IC_50_ indicated stronger effect of inhibition. Acarbose was used as positive control with IC_50_ value at 3.09 ± 0.14 (Figure 1). As a result, except for samples kelp and garlic, all the sample extracts had positive inhibitory activity when the concentration was <50 mg/mL. But the IC_50_ could not be obtained for apricot and eggplant because their inhibitory activities were not dose‐dependent. Moreover, for samples like watermelon, celery, and wax gourd, their IC_50_ values were too large (more than 500 mg/mL) to be calculated. Furthermore, lychee (*Lychee chinensis*) exhibited the strongest *α*‐glycosidase inhibitory activity with IC_50_ value at 10.4 mg/mL which was the smallest one among all samples. Besides, blueberry and plum also showed strong inhibitory effect as the IC_50_ were 13.0 and 10.9 mg/mL, respectively (Figure 2). There were no significantly (*P *<* *0.05) different of IC_50_ values among lychee, blueberry, and plum. In addition, the inhibitory activity between lemon pulp and peel was significantly different (*P < *0.05). At each concentration, lemon pulp showed a higher inhibitory effect and had a lower IC_50_ (18.5 mg/mL) than lemon peel (IC_50_ = 31.1 mg/mL). The least effective one against *α*‐glycosidase owned to bitter gourd with an IC_50_ value at 336.7 mg/mL.

**Table 2:** *α*‐ Glycosidase and aldose reductase inhibitory activities of sample extracts

Samples	*α*‐Glycosidase		Aldose reductase	
	Activity	IC_50_ (mg/mL)	Activity	IC_50_ (mg/mL)
Apricot			P	11.3 ± 0.02^g^
Lychee	P	10.4 ± 0.53^h^	P	NA
Blueberry	P	13.0 ± 0.12^h^	P	16.8 ± 0.50^f^
Plum	P	10.9 ± 0.23^h^	P	27.0 ± 0.54^c^
Kiwi	P	42.7 ± 0.26^c,d^	P	18.5 ± 0.28^e^
Lemon pulp	P	18.5 ± 0.36^g,h^	P	11.3 ± 0.02^g^
Lemon peel	P	31.1 ± 2.51^d,e,f^	P	3.63 ± 0.03^j^
Pear	P	42.2 ± 0.90^c,d^	P	28.5 ± 0.47^b^
Wolfberry	P	26.1 ± 1.78^e,f,g^	P	10.2 ± 0.07^h^
Watermelon	P	NA	P	NA
Lettuce	P	51.4 ± 0.96^c^	P	26.2 ± 0.59^c,d^
Cucumber	P	114.6 ± 4.53^b^	P	25.5 ± 0.34^d^
Red onion	P	22.0 ± 1.26^f,g,h^	P	NA
Bitter gourd	P	336.7 ± 22.66^a^	P	8.55 ± 0.24^i^
Eggplant	N	NA	P	8.06 ± 0.14^i^
Celery	P	NA	P	17.4 ± 0.61^f^
Kelp	P	NA	N	NA
Wax gourd	P	NA	P	36.4 ± 1.30^a^
Garlic	P	NA	P	NA
Tomato	P	36.88 ± 1.92^d,e^	P	29.1 ± 0.67^b^
Acarbose (positive control, *α*‐ Glycosidase inhibitor)	P	3.09 ± 0.14	**–**	**–**

Data are expressed as mean ± standard deviation (*n* = 3). P means the positive dose‐dependent inhibitory activity, whereas N refers to nondose dependency. NA means the IC_50_ is not available to obtain for the non‐dose‐dependent sample extracts or IC_50_ is higher than 500 for the dose‐dependent sample extracts. IC_50_ values of selected samples marked by the same letters within same column are not significantly different (*P *<* *0.05).

### Aldose reductase inhibitory effects of fruits and vegetables

**Figure 3:**
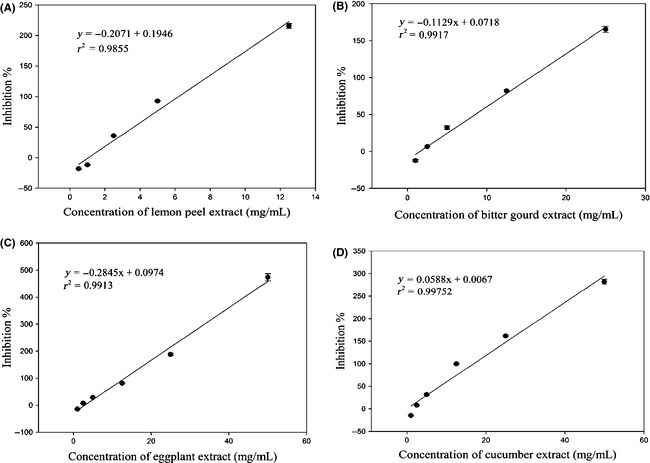
Dose‐dependent aldose reductase inhibitory activity of lemon peel (A), bitter gourd (B), eggplant (C), and cucumber (D).

**Figure 4:**
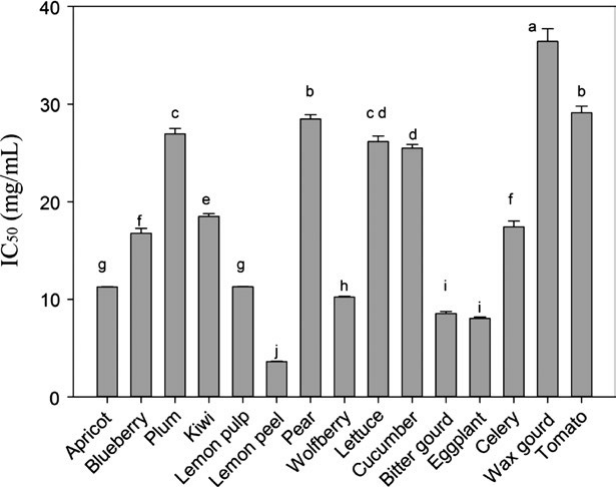
Dose‐dependent aldose reductase inhibitory activity of fruits and vegetables extracts. Figures in the graph were IC_50_ (mg/mL) (*P *<* *0.05).

The protein content of porcine aldose reductase was 3.42 mg BSA/mL and the enzyme activity was 0.95 U/mL. Sample extracts were prepared by dissolving in 5% of DMSO and subjected to AR inhibitory assay. IC_50_ of each sample was calculated, which also indicated the effectiveness of inhibitory activity (Figures 3, 4). AR inhibitory activity potentially showed antidiabetic activity which had been explained in the introduction part. The result showed that lemon peel had the highest AR inhibitory activity (IC_50_ = 3.63 mg/mL), followed by eggplant with an IC_50_ value at 8.06 mg/mL which was not significantly (*P *<* *0.05) different from that of bitter gourd (IC_50_ = 8.55 mg/mL) (Figure 3). Wax gourd had the weakest inhibitory activity (IC_50_ = 36.4 mg/mL) among the selected samples with positive inhibitory effects. Tomato and pear also demonstrated weak inhibitory activity with IC_50_ values at 29.1 mg/mL and 28.5 mg/mL. Garlic had no inhibitory activity as its inhibition rate (−45.7%) was negative at concentration of 50 mg/mL. Lychee, watermelon, and red onion showed dose‐dependency inhibition against AR, but the inhibitory activities were low because their IC_50_ was higher than 500 mg/mL, which failed to be calculated. The inhibitory activities of kelp against AR were positive at concentration of 2.5 mg/mL or higher, and were negative when concentration was 1 mg/mL, but its inhibitory activity was not dose‐dependent which marked as N in Table 2.

### Antioxidant activities of fruits and vegetables

#### Total phenolic content and total flavonoids content of samples

Total phenolic content results (Table 3) were expressed as mg GAE/g sample extract, and lemon peel had the highest TPC with a value at 34.2 ± 0.16 mg GAE/g sample extract, next came wolfberry (26.6 ± 0.14 mg GAE/g sample extract). These two were obviously higher than other samples. Kelp had the lowest TPC (1.14 ± 0.04 mg GAE/g sample extract). As for TFC (Table 3), it was presented in mg CE/g sample extract. Plum had the largest TFC (15.2 ± 0.04 mg CE/g sample extract) and apricot was the second one with TFC value at 14.7 ± 0.60 mg CE/g sample extract. The total flavonoids contents for sample pear, watermelon, kelp, wax gourd and garlic were less than 1 mg CE/g sample extract. Among them, garlic had the lowest TFC (0.25 ± 0.05 mg CE/g sample extract).

**Table 3:** Total phenolic content, total flavonoid, and DPPH free radical scavenging activities of sample extracts

Sample name	Total phenolic content (mg GAE/g sample extract)	Total flavonoid content (mg CE/g sample extract)	DPPH free radical scavenging activity IC_50_ (mg/mL)
Apricot	16.7 ± 0.09^d^	14.7 ± 0.60^b^	4.97 ± 0.05^j^
Lychee	3.32 ± 0.08^o^	1.36 ± 0.08^m^	29.5 ± 0.10^d^
Blueberry	11.6 ± 0.07^h^	5.02 ± 0.08^f^	6.24 ± 0.1^i^
Plum	19.9 ± 0.17^c^	15.2 ± 0.04^a^	5.15 ± 0.03^j^
Kiwi	13.6 ± 0.08^f^	2.10 ± 0.07^k^	7.15 ± 0.01^h^
Lemon pulp	13.0 ± 0.10^g^	4.03 ± 0.06^h^	15.2 ± 0.05^f^
Lemon peel	34.2 ± 0.16^a^	11.19 ± 0.04^c^	8.54 ± 0.07^l,g^
Pear	2.80 ± 0.03^p^	0.99 ± 0.03^n^	NA
Wolfberry	26.6 ± 0.14^b^	6.14 ± 0.02^e^	8.80 ± 0.08^g^
Watermelon	2.12 ± 0.12^q^	0.26 ± 0.05^q^	NA
Lettuce	7.94 ± 0.07^j^	1.72 ± 0.05^l^	NA
Cucumber	5.60 ± 0.04^l^	2.80 ± 0.04^i^	NA
Red onion	6.45 ± 0.04^k^	0.70 ± 0.02^o^	86.6 ± 0.64^b^
Bitter gourd	15.8 ± 0.07^e^	8.59 ± 0.08^d^	8.53 ± 0.38^l,g^
Eggplant	9.00 ± 0.06^i^	4.38 ± 0.06^g^	15.4 ± 0.09^f^
Celery	4.17 ± 0.03^n^	2.28 ± 0.04^j^	103.1 ± 0.68^a^
Kelp	1.14 ± 0.04^r^	0.49 ± 0.04^p^	43.7 ± 0.89^c^
Wax gourd	3.35 ± 0.02^o^	0.26 ± 0.05^q^	43.9 ± 1.18^c^
Garlic	2.67 ± 0.04^p^	0.25 ± 0.05^q^	NA
Tomato	4.94 ± 0.04^m^	1.03 ± 0.04^n^	25.9 ± 0.5^e^

Data are expressed as mean ± standard deviation (*n* = 3). The DPPH scavenging capacity was indicated by IC_50_ (mg/mL). NA means not available to obtain. The values marked by the same letters within same column are not significantly different (*P *<* *0.05).

#### DPPH free radical scavenging activity of samples

The DPPH scavenging capacities of the sample extracts were presented with IC_50_ value (mg/mL) in Table 3, and the smaller the IC_50_ value the higher the scavenging capacities. Except for pear, watermelon, lettuce, cucumber, and garlic, other samples showed positive DPPH free radical scavenging activities, and among them, apricot exhibited the strongest activity as its IC_50_ was the lowest one (4.97 ± 0.05 mg/mL), plum had an IC_50_ value at 5.15 ± 0.03 mg/mL which was the second effective one. Blueberry was the third effective one with IC_50_ value at 7.15 ± 0.01 mg/mL. For vegetable samples, their IC_50_ values ranged from 8.53 mg/mL to 101.1 mg/mL, in which bitter gourd presented remarkable free radical scavenging activity among vegetables. Furthermore, celery had the highest IC_50_ (103.1 ± 0.68 mg/mL), thus the lowest DPPH free radical scavenging activity. Generally, fruit samples demonstrated better antioxidant activity in terms of DPPH free radical scavenging activity than vegetable samples.

## Discussion

### *α*‐Glycosidase inhibitory activities of selected fruits and vegetables

This study proved that some of the ethanol extract from selected samples possessed *α*‐glycosidase inhibitory activities (Table 2). According to Bischoff (1995), the enzyme *α*‐glycosidase is responsible for the absorption of digested glucose from polysaccharide in small intestine. Thus, the inhibitory activity of *α*‐glycosidase would prevent the uptake of glucose which subsequently restrains the increase in blood sugar. The current results showed that nine fruits of 10 selected fruit samples, five vegetables of 10 selected vegetable samples presented dose‐dependent *α*‐glycosidase inhibitory activities. By comparing the *α*‐glycosidase inhibitory activities of selected samples in terms of their IC_50_ value (mg/mL), we found that lychee exhibited the strongest inhibitory activity with an IC_50_ of 10.4 ± 0.53 mg/mL. Lychee is one of the most popular exotic fruits in China. Rare studies reported the antidiabetic properties of lychee pulp, but Ren et al. (2011) stated that two flavanone compounds isolated from lychee seeds exhibited strong *α*‐glycosidase inhibitory activity, and another research reported that lychee tea could regulate blood glucose level in rats (Edel et al. 2006). Thus, the results from this study identified the potential antidiabetic function of lychee pulp. Besides, blueberry and plum also possessed inhibition against *α*‐glycosidase, with IC_50_ of 13.0 mg/mL and 10.9 mg/mL, respectively. Plum was the only sample that had a positive inhibitory activity even at low concentration of 1 mg/mL (the inhibition% was 26.9 ± 0.04%). Plum was the second effective one against *α*‐glycosidase when comparing the IC_50_ of the selected samples. According to Table 4, plum contained the highest amount of total flavonoids (15.2 mg CE/g). The result supported the finding of Kim et al. (2003) who proved that plums were good source of dietary flavonoids, which can also be regarded as the *α*‐glycosidase inhibitors (Gao and Kawabata 2005). As for the blueberry, its inhibitory effectiveness against *α*‐glycosidase has been reported in some studies (Wang et al. 2012). Some other fruits samples like kiwi (IC_50_ = 42.7 mg/mL), lemon pulp (IC_50_ = 18.5 mg/mL), lemon peel (IC_50_ = 31.1 mg/mL), pear (IC_50_ = 42.2 mg/mL), and wolfberry (IC_50_ = 26.1 mg/mL), also presented positive *α*‐glycosidase inhibitory activity. For the vegetable samples, the potential antidiabetic activity (IC_50_ value) in terms of the inhibition against *α*‐glycosidase of them ranked as follow: Red onion (22.0 mg/mL) > tomato (36.9 mg/mL) > lettuce (51.4 mg/mL) > cucumber (114.6 mg/mL) > bitter gourd (336.7 mg/mL). By comparing with the positive control (acarbose) used in the study which had an IC_50_ value at 3.09 ± 0.14 mg/mL, the IC_50_ values of all the selected samples were higher than acarbose. Thus, directly eating the fruits or vegetables instead using drugs is not recommended, but the active constituents in the function samples can be extracted out for further study.

**Table 4:** Correlation between *α*‐glycosidase and aldose reductase inhibitory activities together antioxidant activity

Correlation Coefficient (*r* ^2^)	*α*‐Glycosidase inhibitory activity (IC_50_, mg/mL)	Aldose reductase inhibitory activity (IC_50_, mg/mL)	Total phenolic content (mg GAE/g)	Total flavonoid content (mg CE/g)	DPPH free radical scavenging activity (IC_50_, mg/mL)
*α*‐Glycosidase inhibitory activity (IC_50_, mg/mL)	–				
Aldose reductase inhibitory activity (IC_50_, mg/mL)	0.115^a^	–			
Total phenolic content (mg GAE/g)	0.078	0.435^a^	–		
Total flavonoid content (mg CE/g)	0.163^a^	0.556^a^	0.638^a^	–	
DPPH free radical scavenging activity (IC_50_, mg/mL)	0.004	0.010	0.354^a^	0.379^a^	–

Data in the column are the correlation coefficients between two variables. Pearson's correlation test was conducted to determine the linear correlations among variables.

a Correlation is significant at 0.05 level.

### Aldose reductase inhibitory activities of selected fruits and vegetables

The result from aldose reductase inhibition test can be used to determine the potential antidiabetic effects of selected food samples, as aldose reductase's participation of polyol pathway assist the formation and accumulation of sorbitol which was considered as the cause of some diabetic complications (Kador 1988). In this study, the aldose reductase inhibitory effects of the ethanol extracts from selected samples showed a different picture of the effectiveness.

Differing from the *α*‐glycosidase inhibitory test, most selected samples exhibited a dose‐dependent inhibitory activity against aldose reductase and their 50% inhibitory concentration ranged from 3.63 mg/mL to 36.4 mg/mL. Among these samples, the lemon peel demonstrated the highest aldose reductase inhibitory effect, with the lowest IC_50_ of 3.63 mg/mL. Lemon peel was more effective than lemon pulp (IC_50_ = 11.3 mg/mL) in the inhibition against aldose reductase. On the contrary, the pulp had a stronger *α*‐glycosidase inhibitory activity than peel. Rare studies investigated the aldose reductase inhibitory activity of lemon peel and pulp. The findings in this study showed that lemon peel had more TPC (34.2 mg GAE/g), TFC (11.2 mg CE/g) and better DPPH scavenging activity (IC_50_ = 8.54 mg/mL) than pulp. It can be used as a clue for future study. Eggplant and bitter gourd also demonstrated strong aldose reductase inhibitory activity. Besides there was no significant difference (*P *<* *0.05) between their 50% inhibitory concentration value which were 8.06 mg/mL and 8.55 mg/mL, respectively. Eggplant did not present a positive inhibitory effect against *α*‐glycosidase but it was the second strongest aldose reductase inhibitor. Bitter gourd in this study did not perform well in *α*‐glycosidase inhibitory assay, but its aldose reductase inhibitory activity, as well as the total flavonoids content was ranked at the fourth among 20 selected samples. Vijayalakshmi et al. (2009) suggested that bitter gourd had potential oral hypoglycemic effect and could be used as the functional food because of its antidiabetic effect. The possible mechanisms of bitter gourd which related to the antidiabetic effects had been identified including the stimulation of peripheral and skeletal muscle glucose utilization (Akhtar et al. 2011) and inhibition of glucose uptake (Uebanso et al. 2007). Based on current results, aldose reductase inhibitory activity of bitter gourd would be another possible mechanism. The hypoglycemic effects of the selected samples need further research to support.

Although the IC_50_ values are higher than other reported compounds, it is reasonable that the samples used in the study are crude extracts from foods. One of objectives of this study is to identify active samples which have potential to be further studied. Samples with relative lower IC_50_ values may contain active compounds with low IC_50_ value, the samples with relative lower IC_50_ values deserve to be further studied.

### Antioxidant activities of selected fruits and vegetables

The antioxidant activity and phenolic contents of the selected samples, including total phenolic content, total flavonoids content, and DPPH free radical scavenging activity were tested. According to Madhu and Devi (2000), antioxidant can lower the oxidative stress in diabetes. In this study, lemon peel was found to contain the highest total phenolic content (34.2 ± 0.16 mg GAE/g) and the third highest total flavonoids content (11.19 ± 0.04 mg CE/g). Comparing with the lemon peel, lemon pulp contained a lower TPC and TFC and less effective DPPH free radical scavenging activity. There was little information regarding antioxidant activities of lemon peel and pulp, as well as their antidiabetic effects currently. Wolfberry with the second highest total phenolic content (26.6 ± 0.14 mg GAE/g) also exhibited remarkable aldose reductase inhibitory activity (IC_50_ = 10.2 ± 0.07 mg/mL). The hypoglycemic activity of wolfberry had not been identified yet.

### Correlation analysis

Based on the result shown in Table 4, DPPH free radical scavenging activity in dose‐dependent manner was found in 15 selected samples of 20. The DPPH scavenging activity of selected samples in terms of 50% inhibitory concentration ranged from 4.97 ± 0.05 mg/mL to 103.1 ± 0.68 mg/mL, whereas apricot was identified as the most effective one and celery was found as the least effective one (the highest IC_50_). Apricot contained the second highest TFC in this study. In this study, apricot also demonstrated good AR inhibitory activity. Apricot was reported rich in antioxidants such as phenolics including chlorogenic and neochlorogenic acids, (+)‐catechin, (‐)‐epicatechin, and rutin (Radi et al. 1997).

Total phenolic content and total flavonoids content exhibited positive correlation, with the correlation coefficient (*r* ^2^) equaled to 0.638 (*P *<* *0.05). Moreover, both TPC and TFC showed a negative correlation with the IC_50_ value of DPPH free radical scavenging activities, but TFC carried a better correlation (*r* ^2^ = 0.379, *P *<* *0.05) with DPPH than that of TPC with DPPH (*r* ^2^ = 0.354, *P *<* *0.05). Furthermore, the correlation analyses indicated that there were positive correlations between total flavonoids content and *α*‐glycosidase inhibitory activity (*r* ^2^ = 0.163, *P *<* *0.05, Table 4). Previous studies found that the flavonoids (Gao and Kawabata 2005), anthocyanins (Matsui et al. 2001), and tannin (Huang et al. 2012) content contributed to *α*‐glycosidase inhibitory activity which can support the result of this study.

There was no correlation between *α*‐glycosidase inhibitory activity and aldose reductase inhibitory activity. Many selected samples such as most vegetables did not demonstrate an inhibitory activity against *α*‐glycosidase, but were found to have a dose‐dependent inhibitory activity against aldose reductase. In the correlation analyses, we found that, there were positive correlations between total flavonoids content (TFC) and aldose reductase inhibitory activity (*r* ^2^ = 0.556, *P *<* *0.05, Table 4). The same result was found between TPC and aldose reductase inhibitory activity with *r* ^2^ at 0.435 (*P *<* *0.05). The previous study proved that flavonoids like quercetin, reyneutrin, quercitrin, isoquercitrin, and avicularin had strong inhibitory activity against aldose reductase (Lim et al. 2001).

In general, lemon peel exhibited the greatest antidiabetic activity based on its aldose reductase inhibitory activity, as well as its highest total phenolic content and a remarkable DPPH free radical scavenging activity among the samples. Other samples like apricot, lychee, blueberry, and plum also demonstrated significant potential antidiabetic activity in terms of the effectiveness against *α*‐glycosidase and aldose reductase. On the contrary, watermelon and garlic showed negative inhibitory activity in both *α*‐glycosidase and aldose reductase inhibition assay. In addition, fruits were more effective in the inhibition against *α*‐glycosidase than vegetables, whereas two vegetables samples: eggplant and bitter gourd exhibited good aldose reductase inhibitory activity.

## Conclusions

In conclusion, this study has analyzed the potential antidiabetic effects of the selected foods such as apricot, which exhibited significant aldose reductase inhibitory activity and DPPH free radical scavenging activity. Interesting finding was that lemon peel, the nonedible part, was found more effective against aldose reductase and contained higher antioxidants than lemon pulp. Samples like watermelon, which failed to inhibit the activity of *α*‐glycosidase and aldose reductase also presented the low antioxidant profile. This study also identified the relationship between antioxidant activity and antidiabetic effect. Based on the result obtained in this study, the fruits and vegetables extracts with antidiabetic potential would be further purified and studied to demonstrate significant effect in the treatment of diabetes mellitus.

## Conflict of Interest

None declared.
